# Oral Administration of Si-Based Agent Attenuates Oxidative Stress and Ischemia-Reperfusion Injury in a Rat Model: A Novel Hydrogen Administration Method

**DOI:** 10.3389/fmed.2020.00095

**Published:** 2020-03-20

**Authors:** Masataka Kawamura, Ryoichi Imamura, Yuki Kobayashi, Ayumu Taniguchi, Shigeaki Nakazawa, Taigo Kato, Tomoko Namba-Hamano, Toyofumi Abe, Motohide Uemura, Hikaru Kobayashi, Norio Nonomura

**Affiliations:** ^1^Department of Urology, Osaka University Graduate School of Medicine, Suita, Japan; ^2^The Institute of Scientific and Industrial Research, Osaka University, Ibaraki, Japan; ^3^Department of Nephrology, Osaka University Graduate School of Medicine, Suita, Japan

**Keywords:** silicon, ischemia reperfusion injury, hydrogen, oxidative stress, kidney, rat

## Abstract

Organ ischemia-reperfusion injury (IRI), which is unavoidable in kidney transplantation, induces the formation of reactive oxygen species and causes organ damage. Although the efficacy of molecular hydrogen (H_2_) in IRI has been reported, oral intake of H_2_-rich water and inhalation of H_2_ gas are still not widely used in clinical settings because of the lack of efficiency and difficulty in handling. We successfully generated large quantities of H_2_ molecules by crushing silicon (Si) to nano-sized Si particles (nano-Si) which were allowed to react with water. The nano-Si or relatively large-sized Si particles (large-Si) were orally administered to rats with renal IRI. Animals were divided into four groups: sham, IRI, IRI + nano-Si, and IRI + large-Si. The levels of serum creatinine and urine protein were significantly decreased 72 h following IRI in rats that were administered nano-Si. The levels of oxidative stress marker, urinary 8-hydroxydeoxyguanosine were also significantly decreased with the nano-Si treatment. Transcriptome and gene ontology enrichment analyses showed that the oral nano-Si intake downregulated the biological processes related to oxidative stress, such as immune response, cytokine production, and extrinsic apoptotic signaling pathway. Alterations in the regulation of a subset of genes in the altered pathways were validated by quantitative polymerase chain reaction. Furthermore, immunohistochemical analysis demonstrated that the nano-Si treatment alleviated interstitial macrophage infiltration and tubular apoptosis, implicating the anti-inflammatory and anti-apoptotic effects of nano-Si. In conclusion, renal IRI was attenuated by the oral administration of nano-Si, which should be considered as a novel H_2_ administration method.

## Introduction

Ischemia-reperfusion injury (IRI), which is unavoidable in organ transplantation, is severely detrimental to renal graft function and survival. Prolonged time of cold ischemia during renal transplantation is associated with delayed graft function and decreased long-term graft survival (1, 2). One of the major events in ischemia reperfusion is the generation of cytotoxic oxygen radicals (3), increases in which lead to cellular injury by inducing DNA damage, protein oxidation, lipid peroxidation, and apoptosis (4).

Since the discovery of the selective antioxidant properties of molecular hydrogen (H_2_) in 2007, multiple studies have shown its beneficial effects in diverse animal oxidative stress models *in vivo* (5–7). H_2_ treatment has been shown to abrogate ischemia-reperfusion following warm and cold ischemia and has been identified as a potential therapy in improving kidney transplantation outcomes (8). Studies have explored several delivery systems for H_2_ administration, including inhalation, oral intake of H_2_-rich water, injection of H_2_-rich saline, and direct incorporation (9). Nevertheless, to our knowledge, these methods are not widely used in clinical settings because of the lack of efficacy and difficulty in handling.

We have recently reported that nano-sized silicon (Si) particles (nano-Si) react with water in the pH range between 7.0 and 8.6, generating a large amount of H_2_ (10). We have found that the H_2_ generation rate strongly depended on the crystallite size of particles and pH of the water reacting with Si. In water with a pH of 8.0, nano-Si with a median diameter of 9.6 nm could generate up to 55 mL/g H_2_ within 1 h, which corresponds to that contained in ~3 L saturated H_2_-rich water. Si and its oxide are non-poisonous materials and are therefore considered as appropriate for medical applications (11).

We hypothesized that oral administration of a diet containing nano-Si would react with water in the intestinal tract where alkaline pancreatic juices are secreted, thereby leading to the generation of H_2_ and suppression of reactive oxygen species. Therefore, in the current study we investigated the potential of nano-Si in mitigating IRI *in vivo* and reducing oxidative stress and related biological processes. To that end, we administered a diet containing nano-Si or larger-sized Si particles (large-Si) with a minimum diameter of 1 μm in rats with renal IRI.

## Materials and Methods

### Animals

All experiments were performed in male Sprague-Dawley rats weighing 170–190 g that were purchased from SLC Japan (Shizuoka, Japan) and maintained at the Institute of Experimental Animal Sciences of Osaka University Medical School. All animal studies were approved by the Osaka University Animal Research Committee and according to relevant regulatory standards.

### Si Particles Containing Feed

As normal diet, we used AIN93M (Oriental Yeast Co., Ltd., Tokyo, Japan). In addition, we made special Si-based agent containing 1.0 wt.% nano-Si or large-Si particles in AIN93M, respectively. Before animal experiments, we examined the hydrogen production from the agent containing 1.0 wt.% nano-Si particles and water using a sensor gas chromatograph, SGHA-P2-A (FIS Inc., Hyogo, Japan).

### Experimental Protocol

Experimental groups (*n* = 6 per group) were as follows: (i) sham operation (sham group), (ii) normal diet with IRI (IRI group), (iii) nano-Si-based agent diet with IRI (IRI + nano-Si group), and (iv) large-Si-based agent diet with IRI (IRI + large-Si group). The animals in the sham and IRI groups were fed a normal diet. The animals in the IRI + nano-Si and IRI + large-Si groups were fed a Si-based agent containing 1.0% nano-Si and large-Si in a diet, respectively. The Si-based agent was initiated at 6 weeks of age. Renal IRI or sham surgery was performed at 7 weeks of age, as previously described (12). Briefly, rats were anesthetized with isoflurane and placed on a heating pad to maintain body temperature during surgery. A midline abdominal incision was made, and left renal pedicles were isolated and clamped for 60 min. Complete reperfusion was visually confirmed after the clamp removal. After reperfusion of the left kidney, right nephrectomy was performed and the surgical wound was sutured. Sham surgery was performed in an identical fashion with the exception of renal pedicle clamping. The rats were euthanized 72 h after reperfusion, and blood, urine, and kidney samples were obtained.

### Measurement of H_2_ Concentration Diffused From Whole Blood Samples

After 1-week administration of the normal diet or the diet containing 1.0% nano-Si or large-Si, 200 μL whole blood samples were immediately collected into 20-mL glass tubes. The glass tubes were filled with fresh air before putting the samples, and the tops were covered using screw tops with attached silicon caps. Next, the tubes with the samples were placed for 30 min at room temperature, and 2 mL gas (vapor) were aspirated from the tube and injected into a sensor gas chromatography device, SGHA-P2-A. The H_2_ concentrations in blood samples were adjusted to the H_2_ concentrations in air which fluctuated daily.

### Histological Analysis

For histological evaluation, the frozen renal sections extracted from rats were embedded in 4% paraformaldehyde. Histological sections were stained with hematoxylin and eosin and assessed by a transplant pathologist blinded to the conditions. Tubular damage was graded based on the following scale ranging from 0 to 5 by estimating the percentage of tubules in the corticomedullary junction showing epithelial necrosis, loss of nuclei, or cast formation: 0, none; 1, <10%; 2, 10–25%; 3, 26–50%; 4, 51–75%; and 5, >75%.

### Measurement of Oxidative Damage

8-hydroxy-2′-deoxyguanosine (8-OHdG), a product of oxidative DNA damage, is widely used as a marker of oxidative stress. We measured urinary 8-OHdG levels using an enzyme-linked immunosorbent assay kit (Japan Institute for the Control of Aging, Shizuoka, Japan). A monoclonal antibody against 8-OHdG and urine samples or standards were added to microtiter plates precoated with 8-OHdG. An enzyme-labeled secondary antibody was then added to the plates and allowed to bind to the monoclonal 8-OHdG antibody on the coated plates. The unbound enzyme-labeled secondary antibody fraction was removed by washing, and a chromatic substrate was added for color development. The color reaction was terminated, and the absorbance at 450 nm was measured by a microplate reader. Malondialdehyde (MDA) is used as an indicator of free radical-mediated lipid peroxidation. We measured serum malondialdehyde levels using the OxiSelect TBARS assay kit (Cell Biolabs, San Diego, CA, USA). Briefly, 100 mL of serum samples or a solution with a known MDA concentration were mixed with 100 mL SDS lysis solution and 250 mL thiobarbituric acid solution and heated at 95°C in a water bath for 1 h. After cooling, the samples were centrifuged to collect the supernatants, which were mixed with n-butanol followed by centrifugation. The butanol fractions were transferred to 96-well microplates and the change in color was determined at 532 nm using a spectrophotometric plate reader (ELx808, Bio Tek Instruments).

### RNA Microarray

Total RNA from postoperative frozen kidneys was isolated by homogenization followed by the RNeasy Plus universal kit (Qiagen, Hilden, Germany). RNA was treated with DNAse and reverse transcribed to cDNA by the PrimeScript RT reagent kit (Takara Bio, Shiga, Japan). RNA quality was assessed using a 2100 Bioanalyzer (Agilent Technologies, Santa Clara, CA, USA) and the RNA 6000 Nano kit (Caliper Life Sciences, CA, USA). The RNA samples isolated from the IRI and IRI + nano-Si groups (*n* = 2 per group) were hybridized to Rat GeneChip Clariom™ S array (Applied Biosystems, CA, USA). The hybridization and microarray scanning procedures were performed according to the Whole Transcript (WT) expression array user guide. After the probe set signal integration and background correction, the files were transferred to the Applied Biosystems Transcriptome Analysis Console software to analyze gene expression patterns. The threshold for upregulated and downregulated genes was set as a fold change of ≥1.5 with a *p* < 0.05.

### Gene Ontology and Pathway Enrichment Analyses

Functional enrichment analysis was performed by Metascape (http://metascape.org) according to the genes assigned to each biological function. The resulting gene ontology terms with a *p* < 0.05 were considered significantly enriched among the differentially expressed genes.

### Quantitative Real-Time Reverse-Transcriptase Polymerase Chain Reaction

Quantitative real-time reverse-transcriptase polymerase chain reaction was performed using TB Green Premix Ex Taq II and a Thermal Cycler Dice Real Time System TP800 (Takara). The primer sequences were as follows: C-C motif chemokine ligand 2 (*Ccl2*): forward: CTATGCAGGTCTCTGTCACGCTTC, reverse: CAGCCGACTCATTGGGATCA; interleukin 6 (*Il6*): forward: ATTGTATGAACAGCGATGATGCAC, reverse: CCAGGTAGAAACGGAACTCCAGA; intercellular adhesion molecule 1 (*Icam1*): forward: TGTATGAACTGAGCAATGTGCAAGA, reverse: CACCTGGCAGCGTAGGGTAA; inducible nitric oxide synthase (*iNos*): forward: CTCACTGTGGCTGTGGTCACCTA, reverse: GGGTCTTCGGGCTTCAGGTTA; tissue inhibitor of metalloproteinase-1 (*Timp1*): forward: CGAGACCACCTTATACCAGCGTTA, reverse: TGATGTGCAAATTTCCGTTCC; phorbol-12-myristate-13-acetate-induced protein 1 (*Pmaip1*): forward: GGAGTGCACCGGACATAACTG, reverse: TGCCGTAAATTCACTTTGTCTCCA; catalase (*Cat*): forward: GAACATTGCCAACCACCTGAAAG, reverse: GTAGTCAGGGTGGACGTCAGTGAA; peroxisome proliferator-activated receptor alpha (*PPARa*): forward: GGCAATGCACTGAACATCGAG, reverse: GCCGAATAGTTCGCCGAAAG; and beta-actin (*Actb*): forward: GGAGATTACTGCCCTGGCTCCTA, reverse: GACTCATCGTACTCCTGCTTGCTG. We used the ^ΔΔ^ cycle threshold technique to calculate the cDNA content of each sample. Target gene signals were normalized to *Actb*. Melting curve analysis showed a single dissociation peak for all polymerase chain reaction gene products, confirming the specificity of the reactions.

### Immunohistochemical Analysis

The terminal deoxynucleotidyl transferase dUTP nick end labeling assay was used to assess apoptosis in frozen renal sections, according to the manufacturer's instructions (Japan Institute for the Control of Aging). In addition, frozen sections were incubated with an antibody against the macrophage surface molecule CD68. Anti-CD68 antibody was bound to a biotinylated secondary antibody which reacted with several peroxidase-conjugated streptavidin molecules using the LSAB + System-HRP kit (Dako, Copenhagen, Denmark). The sections were then visualized with the DAB chromogen. The images were captured using a BZ-X700 (Keyence, Osaka, Japan) microscope and the areas or cells with positive immunohistochemical signals were assessed using the software of the BZ-x700 microscope. In TUNEL staining, the ratio of the number of positive cells to total cells in the entire area including interstitium was calculated, and in the staining for CD68, the ratio of the area of the positive cells to the entire area was calculated.

### Statistical Analysis

GraphPad Prism version 5.0 (GraphPad, San Diego, CA, USA) was used for all statistical analyses. Multiple groups were compared using one-way analysis of variance with Tukey's *post*-*hoc* multiple comparison test. Results were expressed as means ± standard error of the mean. Differences with a *p* < 0.05 were considered to indicate statistical significance.

## Results

### Oral Nano-Si Administration Preserves Renal Function and Reduces Tubular Damage Due to IRI

**Figure 1:**
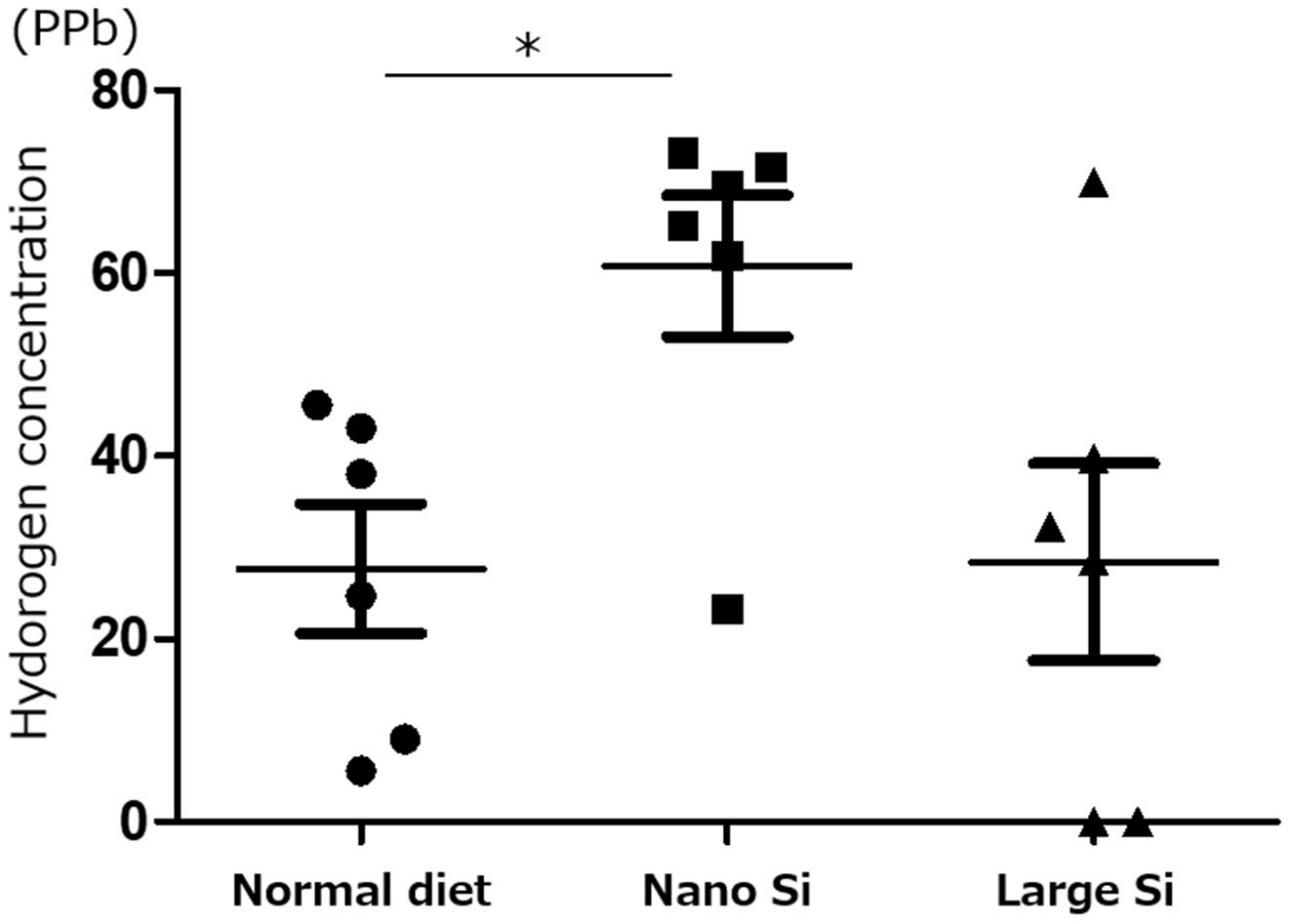
Measurement of hydrogen (H_2_) concentration diffused from whole blood samples using gas chromatography. All H_2_ concentrations were adjusted to the H_2_ concentration in air. Bars represent means ± standard error of the mean. *p < 0.05, *n* = 6.

**Figure 2:**
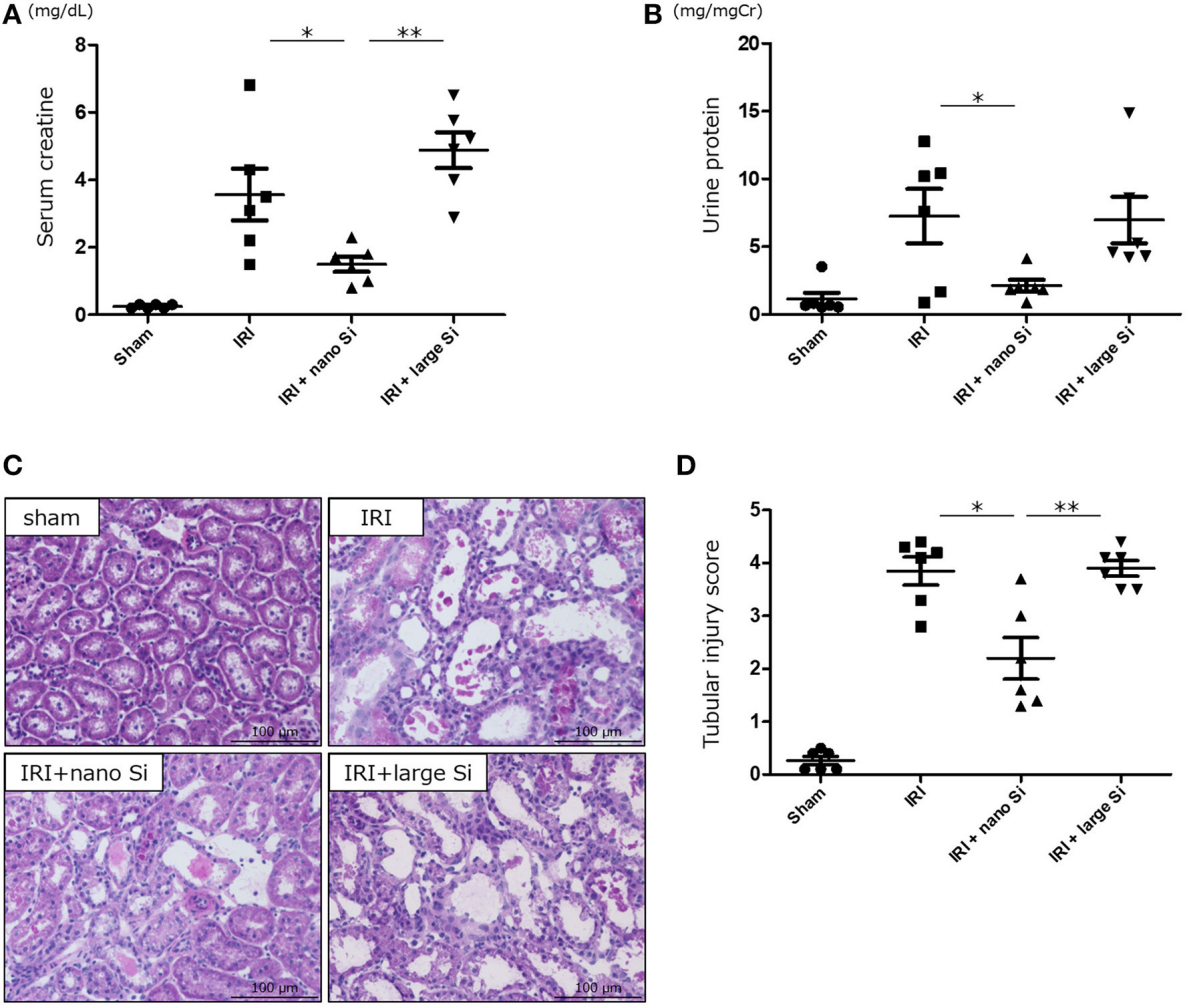
Oral administration of nano-sized silicon (Si) particles (nano-Si) improves renal function and tubular damage following ischemia-reperfusion injury (IRI). Serum creatinine **(A)** and urine protein levels **(B)** at 72 h after surgery for ischemia-reperfusion in animals receiving normal diet (IRI group) or a diet comprising nano-Si (IRI + nano-Si group) or large-sized Si (IRI + large-Si group) as well as those undergoing sham surgery (sham group). **(C)** Representative images of renal sections stained with hematoxylin and eosin at 72 h after surgery in the sham, IRI, IRI + nano-Si, and IRI + large-Si groups. **(D)** Semiquantitative analysis of the images using tubular injury score. Bars represent means ± standard error of the mean. *p < 0.05, **p < 0.01, *n* = 6.

We first confirmed that oral administration of nano-Si could generate H_2_ in rats in the absence of IRI by gas chromatography (Figure 1). Compared with those maintained on a normal diet, the H_2_ concentrations diffused from the whole blood samples were significantly increased in those administered a diet comprising nano-Si but not large-Si. Next, we evaluated renal function by measuring serum creatinine and urine protein levels at 72 h after surgery. As shown in Figures 2A,B, the serum creatinine and urine protein levels were significantly decreased in the IRI + nano-Si group compared with the IRI group whereas there was no significant decrease in either parameter in the IRI + large-Si group compared with the IRI group. Additionally, the structural injury in the renal corticomedullary junction such as epithelial necrosis, loss of nuclei, and cast formation occurred as a result of IRI. Consistent with the detected changes in renal function, the kidneys in the IRI + nano-Si group had significantly less tubular damage than those in the IRI and IRI + large-Si groups, as evidenced by the tubular injury score (Figures 2C,D).

### Effect of Nano-Si Treatment on Oxidative Stress

**Figure 3:**
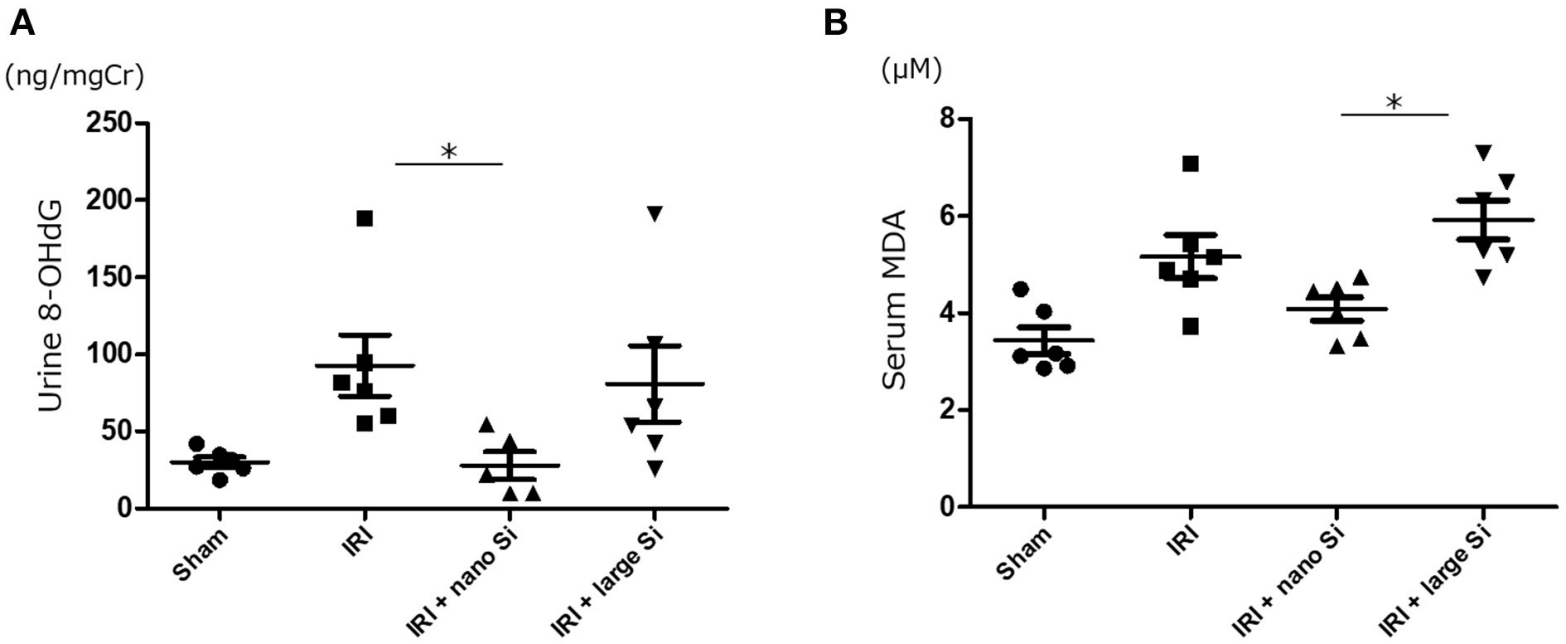
Effect of nano-Si treatment on markers of oxidative stress. **(A)** Urine 8-hydroxy-2'-deoxyguanosine (8-OHdG) and **(B)** serum malondialdehyde (MDA) levels at 72 h after surgery in the sham, IRI, IRI + nano-Si, and IRI + large-Si groups. Bars represent means ± standard error of the mean. *p < 0.05, *n* = 6.

8-OHdG and malondialdehyde are major forms of DNA and lipid damage induced by reactive oxygen species, respectively. Therefore, we next measured the urinary 8-OHdG and serum malondialdehyde levels to assess oxidative stress. There was a significant reduction in oxidative DNA damage assessed by urinary 8-OHdG 72 h after surgery in the IRI + nano-Si group (Figure 3A). Additionally, the serum malondialdehyde levels were significantly decreased in the IRI + nano-Si group compared with the IRI + large-Si group (Figure 3B). Albeit not significant, there was a difference in lipid peroxidation between the IRI and IRI + nano-Si groups.

### Effect of Nano-Si Treatment on Renal Transcriptome and Biological Functions

**Figure 4:**
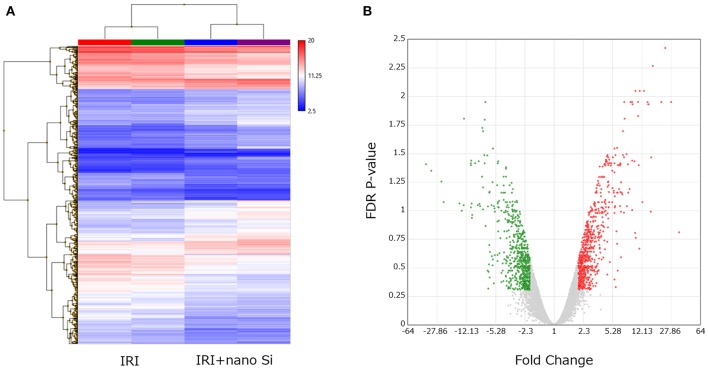
Changes in the renal transcriptome at 72 h after IRI. **(A)** Hierarchical clustering of each sample dataset by renal tissue microarray. **(B)** Volcano plot showing the upregulated and downregulated genes in the kidneys of rats with IRI treated with nano-Si compared to those maintained with a normal diet (fold change, >2.0 or <2.0; *p* < 0.05). *n* = 2.

**Figure 5:**
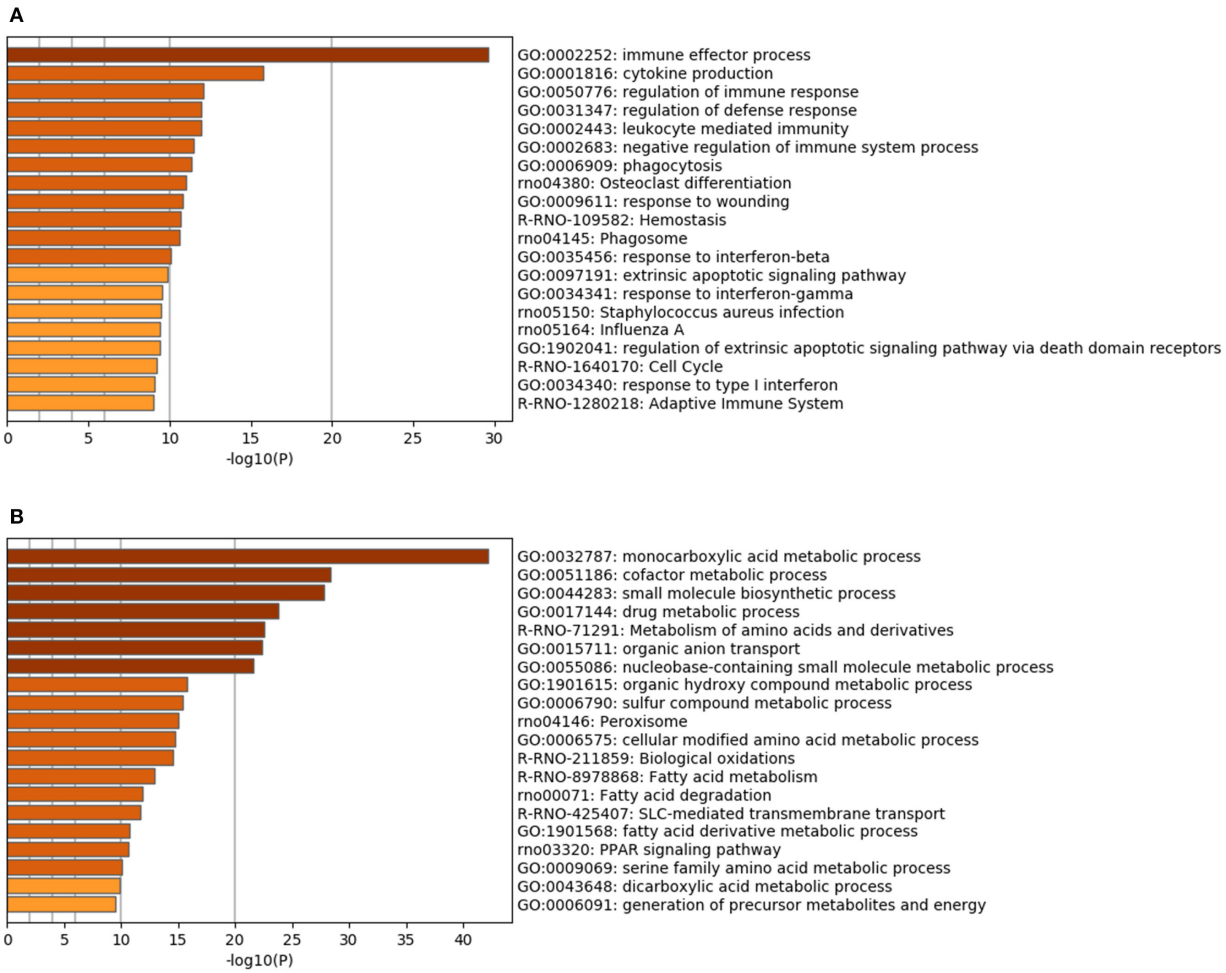
Functional enrichment analysis of the genes modulated with the nano-Si treatment. Top 20 biological processes with upregulated **(A)** and downregulated **(B)** genes, with *p*-values, in the renal tissue samples at 72 h following IRI.

Renal RNA microarray analysis was performed to compare the IRI and the IRI + nano-Si groups for changes in renal transcriptomes of rats with IRI treated with nano-Si (Figure 4A). As shown in Figure 4B, we identified 666 downregulated genes and 703 upregulated genes (Figure 4B). We first performed gene ontology and pathway enrichment analysis of the 666 downregulated genes. Figure 5A lists the top 20 downregulated biological processes with *p* values, including pathways associated with immune response such as cytokine production and phagocytosis as well as the extrinsic apoptotic pathway. The gene ontology and pathway enrichment analysis of the 703 upregulated genes revealed that the top 20 upregulated pathways included primarily the processes related to some kind of amino acid metabolism such as fatty acids (Figure 5B). The biological processes involved in peroxisome were also ranked in the top 20 upregulated pathways.

### Validation of Changes in Gene Expression Levels by Quantitative Polymerase Chain Reaction

**Figure 6:**
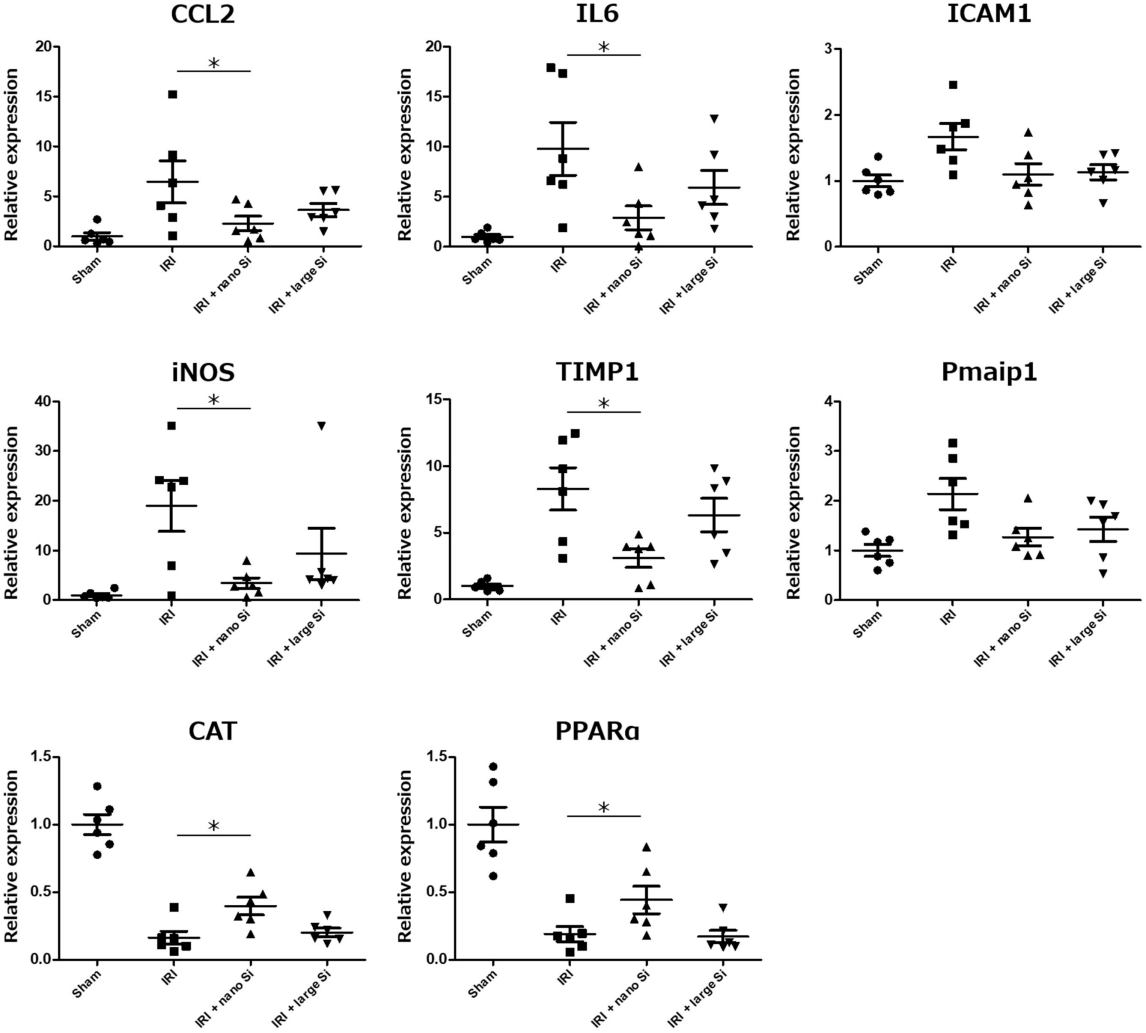
Relative mRNA expression levels of eight genes associated with inflammation, apoptosis, and oxidative stress determined using real-time reverse-transcription polymerase chain reaction in the renal tissue samples at 72 h following surgery in the sham, IRI, IRI + nano-Si, and IRI + large-Si groups. *p < 0.05, *n* = 6.

Real-time reverse-transcription polymerase chain reaction was performed to validate the changes in expression of genes related to the biological processes modulated by nano-Si administration which we identified in the gene enrichment analysis (Figure 6). We assessed the expression levels of pro-inflammatory genes including *Cccl2, Il6*, and *Icam1*; those involved in extrinsic apoptotic signaling pathway including *Timp1* and *Pmaip1*; *iNOS* which is induced by oxidative stress; and those induced by the peroxisome including *Cat* and *PPARa*. Figure 6 shows the fold changes in mRNA levels of the indicated genes in the IRI + nano-Si group compared with the sham group, normalized to *Actb*. Overall, the oral nano-Si treatment of rats with IRI led to significant reductions in the mRNA levels of *Cccl2, Il6, Timp1*, and *iNos* compared with the IRI group. Conversely, the expression levels of *Cat* and *PPARa* were significantly decreased in the kidneys of the IRI group compared with the sham group and increased in the IRI + nano-Si group compared with the IRI group.

### Nano-Si Treatment Alleviates Interstitial Macrophage Infiltration and Tubular Apoptosis

**Figure 7:**
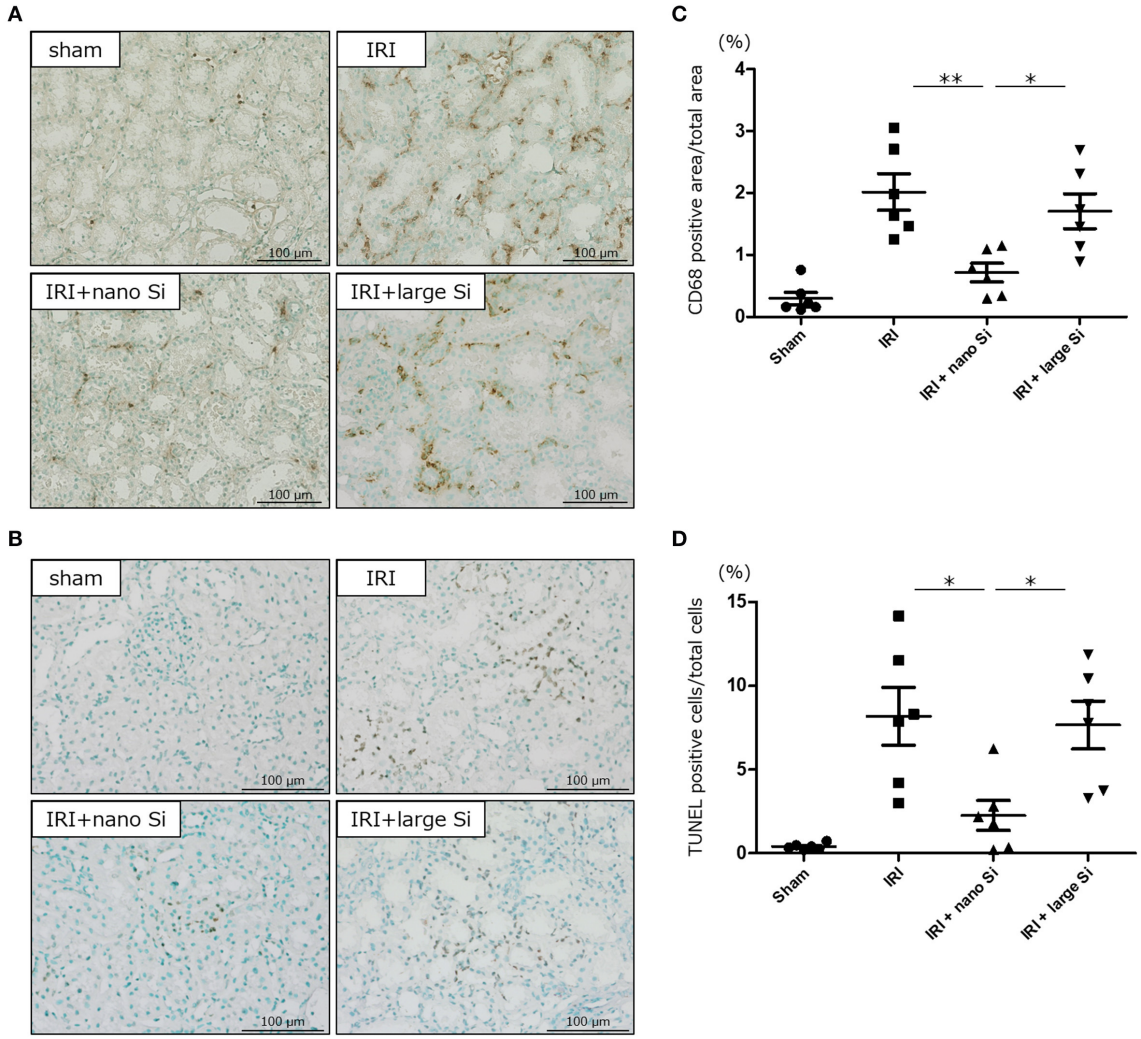
Oral administration of nano-Si particles alleviates interstitial macrophage infiltration and tubular apoptosis. Representative images of **(A)** CD68 staining and **(B)** terminal deoxynucleotidyl transferase dUTP nick end labeling (TUNEL) of the renal tissues at 72 h after surgery in the sham, IRI, IRI + nano-Si, and IRI + large-Si groups. **(C)** Quantitative analysis of the images showing changes in the ratio of CD68-positive areas to the total area of the section. **(D)** Quantitative analysis of the images showing changes in the ratio of TUNEL-positive cells to the total number of cells. Bars represent means ± standard error of the mean. *p < 0.05, **p < 0.01, *n* = 6.

Finally, we evaluated interstitial infiltration of macrophages and tubular apoptosis in the kidneys of rats with IRI that were treated with nano-Si (Figures 7A,B). Immunohistochemical analysis demonstrated that the nano-Si treatment led to a significant reduction in the CD68-positive macrophage infiltration at both the corticomedullary junction and the cortex of kidneys following IRI compared with the sham and the IRI + large-Si groups (Figure 7C). Moreover, the tubular apoptosis observed in the IRI and IRI + large-Si groups was significantly inhibited by the oral nano-Si administration (Figure 7D).

## Discussion

H_2_ is a noble gas that eliminates reactive oxygen species that cause IRI. Studies on the role of H_2_ in biology and medicine have been rapidly expanding since the first report of Ohsawa et al. (5) demonstrating that H_2_ gas displays antioxidant properties that can protect the brain against IRI by selectively neutralizing hydroxyl radicals. Several delivery systems for H_2_ administration that have been explored include inhalation using a ventilator circuit, facemask, or nasal cannula, which has been reported to reduce IRI in the brain (13), heart (14), lungs (15), liver (16), skin (17), and intestines (6). Although inhaled H_2_ gas may act rapidly, this method might be impractical in daily clinical use or unsuitable for continuous consumption for preventive use, because of the difficulty in handling high-pressure H_2_ gas.

In contrast, oral intake of solubilized H_2_ in H_2_-rich water is easy to use as a portable and safe method of delivering molecular H_2_ (18). However, H_2_ in water evaporates over time, and H_2_ might be lost before reaching the stomach or the intestines (9). Administration of H_2_ via injectable H_2_-rich saline can potentially deliver more precise H_2_ concentrations and has been demonstrated to attenuate IRI in brain in neonates (19), heart (20), lungs (21), liver (22), intestines (23, 24), and kidneys (25) in animal models of oxidative stress. Nevertheless, the amount of H_2_ dissolved in solutions is limited: up to 0.8 mM (1.6 mg/L) H_2_ can be dissolved in water under atmospheric pressure at room temperature (26).

We have developed a new strategy to successfully generate large amounts of H_2_ molecules by crushing Si to nano-sized particles and allowing these nanoparticles to react with alkaline water. Si is not an essential nutrient for mammals, and the underlying biological function remains unclear; however, oral Si supplementation for mammals is reportedly harmless (11). We therefore evaluated the efficacy of nano-Si administration in an *in vivo* model of acute renal IRI. Our serological, urinary, and histological analyses showed the deterioration of renal function and damage to tubular epithelial cells following ischemia-reperfusion surgery. In contrast, the administration of nano-Si-containing diet significantly reduced these changes. This benefit of nano-Si is considered to be due to the generation of H_2_ reacting with alkaline water such as that found in intestinal fluids *in vivo*. In support of this hypothesis, the diet containing relatively larger Si particles with poor H_2_ generation efficiency, which was administered following ischemia-reperfusion surgery using the same dosing schedule, did not lead to a similar effect.

Given that H_2_ molecules can mitigate IRI by selectively removing reactive oxygen species that lead to oxidative damage to DNA, lipids, and proteins, we evaluated oxidative stress by monitoring urinary 8-OHdG and serum malondialdehyde levels. 8-OHdG and malondialdehyde, which are peroxidation products of DNA and lipids, respectively, are widely used as oxidative stress markers (27–29). The treatment with nano-Si led to a reduction in the increased urinary 8-OHdG levels due to IRI. Conversely, albeit not reaching statistical significance, we also found that there was a difference in the serum malondialdehyde levels between the IRI and IRI + nano-Si groups.

H_2_ has been shown to exert anti-inflammatory and anti-apoptotic effects by suppressing oxidative stress (30). In fact, specific pathways and modulators underlying these effects have not been fully elucidated. In the present study, we demonstrated that the nano-Si treatment inhibited the infiltration of macrophages into the tubulointerstitium and the upregulated expression of inflammation-related genes such as *Il6* and *Ccl2* in renal tissue. Macrophages are critical early initiators of innate immunity with important roles in inflammation following ischemia-reperfusion (31, 32). Additionally, activated endothelial cells and tubular epithelial cells produce cytokines and chemokines that induce inflammatory cell infiltration. We also demonstrated that the administration of the nano-Si reduced apoptosis in tubular epithelial cells after the induction of ischemia by hypoxia using terminal deoxynucleotidyl transferase dUTP nick end labeling assay to detect early apoptosis based on the presence of DNA fragmentation (33). Overall, these results suggest that the reduction in oxidative stress leads to anti-inflammatory and anti-apoptotic effects by regulating the expression levels of various genes. The proteins encoded by these genes, which may not be primary responders to H_2_, might indirectly act to enable the various beneficial effects of nano-Si.

In conclusion, we demonstrated that the nano-Si treatment protected IRI rat kidneys, at least partially, via the reduction in oxidative stress through oral intake of nano-Si. To our knowledge, these findings are the first evidence for the protective effect with nano-Si in renal IRI. Given our results, the clinical use of nano-Si in kidney allograft donors prior to kidney transplantation should be considered as a means to improve kidney allograft outcomes. Thus, further investigation is needed to establish the feasibility and efficacy of using nano-Si in this way in the clinical setting.

## Data Availability Statement

The datasets generated for this study can be found on ArrayExpress, accession number E-MTAB-8687.

## Ethics Statement

The animal study was reviewed and approved by Osaka University Animal Research Committee.

## Author Contributions

MK and RI designed, performed, and interpreted all the experiments. YK and HK developed and offered the experimental materials. Histological and immunohistochemical sections were assessed by TN-H. AT, SN, and TK participated in the interpretation of data and editing of the manuscript. TA, MU, and NN interpreted the data, edited, and finalized the manuscript.

### Conflict of Interest

The authors declare that the research was conducted in the absence of any commercial or financial relationships that could be construed as a potential conflict of interest.
